# Metabolic connectivity of resting-state networks in alpha synucleinopathies, from prodromal to dementia phase

**DOI:** 10.3389/fnins.2022.930735

**Published:** 2022-08-08

**Authors:** Cecilia Boccalini, Elisa Bortolin, Giulia Carli, Andrea Pilotto, Andrea Galbiati, Alessandro Padovani, Luigi Ferini-Strambi, Daniela Perani

**Affiliations:** ^1^School of Psychology, Vita-Salute San Raffaele University, Milan, Italy; ^2^In Vivo Human Molecular and Structural Neuroimaging Unit, Division of Neuroscience, IRCCS San Raffaele Scientific Institute, Milan, Italy; ^3^Department of Nuclear Medicine and Molecular Imaging, University Medical Center Groningen, University of Groningen, Groningen, Netherlands; ^4^Neurology Unit, Department of Clinical and Experimental Sciences, University of Brescia, Brescia, Italy; ^5^Parkinson’s Disease Rehabilitation Centre, FERB ONLUS, S. Isidoro Hospital, Trescore Balneario, Italy; ^6^Department of Clinical Neuroscience, Sleep Disorders Center, San Raffaele Hospital, Milan, Italy; ^7^Nuclear Medicine Unit, San Raffaele Hospital, Milan, Italy

**Keywords:** metabolic connectivity, large-scale brain networks, alpha-synuclein spectrum, isolated REM sleep behavior disorder, [18F]FDG-PET

## Abstract

Previous evidence suggests that the derangement of large-scale brain networks reflects structural, molecular, and functional mechanisms underlying neurodegenerative diseases. Although the alterations of multiple large-scale brain networks in Parkinson’s disease (PD) and Dementia with Lewy Bodies (DLB) are reported, a comprehensive study on connectivity reconfiguration starting from the preclinical phase is still lacking. We aimed to investigate shared and disease-specific changes in the large-scale networks across the Lewy Bodies (LB) disorders spectrum using a brain metabolic connectivity approach. We included 30 patients with isolated REM sleep behavior disorder (iRBD), 28 with stable PD, 30 with DLB, and 30 healthy controls for comparison. We applied seed-based interregional correlation analyses (IRCA) to evaluate the metabolic connectivity in the large-scale resting-state networks, as assessed by [18F]FDG-PET, in each clinical group compared to controls. We assessed metabolic connectivity changes by applying the IRCA and specific connectivity metrics, such as the weighted and unweighted Dice similarity coefficients (DC), for the topographical similarities. All the investigated large-scale brain resting-state networks showed metabolic connectivity alterations, supporting the widespread involvement of brain connectivity within the alpha-synuclein spectrum. Connectivity alterations were already evident in iRBD, severely affecting the posterior default mode, attentive and limbic networks. Strong similarities emerged in iRBD and DLB that showed comparable connectivity alterations in most large-scale networks, particularly in the posterior default mode and attentive networks. Contrarily, PD showed the main connectivity alterations limited to motor and somatosensory networks. The present findings reveal that metabolic connectivity alterations in the large-scale networks are already present in the early iRBD phase, resembling the DLB metabolic connectivity changes. This suggests and confirms iRBD as a risk condition for progression to the severe LB disease phenotype. Of note, the neurobiology of stable PD supports its more benign phenotype.

## Introduction

Parkinson’s disease (PD), dementia with Lewy bodies (DLB), and multiple system atrophy (MSA) are clinical entities of the alpha-synucleinopathies spectrum, characterized by the abnormal accumulation in the brain of misfolded alpha-synuclein (Goedert et al., 2017). Specifically, PD and DLB are associated with Lewy Bodies (LB) (in the soma) and Lewy neurites (LN) (in neural dendrites) inclusions falling under the umbrella term of LB diseases; instead, MSA is characterized by glial cytoplasmic inclusion (Calo et al., 2016). The isolated REM sleep behavior disorder (iRBD) is considered a prodromal stage of alpha-synucleinopathies with a high risk of progression. The association of iRBD with alpha-synuclein-related neurodegeneration is strongly substantiated by clinical follow-up and neuropathological studies in case series (Schenck, 2019). Specifically, Lewy-type pathology has been observed in the brains of iRBD patients (Boeve et al., 2013; Iranzo et al., 2013, 2014). More than 80% of patients with iRBD convert into PD, PD dementia (PDD), DLB, or, in rarer circumstances, MSA after approximately 14 years of follow-up (St Louis et al., 2017; Galbiati et al., 2019).

Increasing evidence proves that iRBD can be considered a red flag for a severe clinical phenotype characterized by dementia development (Lin and Chen, 2018). Clinical data support that iRBD could be the prodromal manifestation of a diffuse malignant clinical subtype of PD (i.e., PDD/DLB) (Lin and Chen, 2018). RBD prevalence in DLB is 80% against 16–47% in PD (Högl et al., 2018). Moreover, PD patients with RBD (PD-RBD) manifest an aggressive disease course characterized by autonomic dysfunction, visual hallucinations, and also dementia (PDD) (Fereshtehnejad et al., 2017; Lin and Chen, 2018; Pilotto et al., 2019). However, the retrospective nature of iRBD diagnosis is a frequent limit in clinical research. Evaluating the neurobiological changes underlying the prodromal (iRBD) and full-blown LB disease with (PDD/DLB) and without dementia (PD) has crucial relevance to establishing the role of iRBD as a prognostic marker.

In this context, [18F]FDG-PET has shown promising results through different analytical approaches, revealing a common pathological substrate between DLB/PDD and iRBD. Multivariate and univariate approaches revealed a significative occipital hypometabolism in iRBD patients (Wu et al., 2014; Ge et al., 2015; Meles et al., 2018; Carli et al., 2020b). Occipital hypometabolism has been associated with a higher risk of developing dementia in PD (Pilotto et al., 2018) and represents a hallmark metabolic signature of DLB (Caminiti et al., 2019). Thus, the occipital vulnerability in iRBD might represent an early sign of malignant LB disease clinical phenotype characterized by dementia development (DLB/PDD; Wu et al., 2014; Ge et al., 2015; Meles et al., 2018; Carli et al., 2020b). Common pathological substrates between PDD/DLB and iRBD also emerged from brain metabolic connectivity targeting the dopaminergic, noradrenergic, and cholinergic systems, known to be affected by alpha-synuclein aggregations (Uchihara and Giasson, 2016). Specifically, iRBD and DLB share similar cholinergic alterations, and in PD without cognitive deterioration, a limited impairment of this neurotransmission system is present (Carli et al., 2020c). All the above suggest that iRBD might have more biological mechanisms in common with DLB than PD with a stable clinical progression, providing initial biological support to the definition of iRBD as a risk factor for dementia development to LB diseases (Carli et al., 2020b; Galbiati et al., 2021). However, little is known about the alteration of large-scale networks between iRBD and full-blown LB diseases.

Abnormal aggregations of alpha-synuclein cause dysfunctions in synaptic transmission and produce widespread effects on functional connectivity among distant brain regions, thus resulting in alterations of large-scale brain networks (Palop et al., 2006). Thus, in neurodegenerative diseases, a clearer understanding of the underlying abnormal networks could aid the achievement of an effective early diagnosis and the identification of potential treatment targets (Niethammer and Eidelberg, 2012; Schindlbeck and Eidelberg, 2018). Multivariate brain connectivity methods applied to [18F]FDG-PET data emerged in the last decade as innovative and powerful approaches to unraveling the pathophysiology of neurodegenerative diseases (Yakushev et al., 2017; Schindlbeck and Eidelberg, 2018; Sala and Perani, 2019). Even if functional MRI (fMRI) has dominated the brain connectivity scene for decades, it is becoming increasingly clear that fMRI connectivity measures have limited reproducibility, especially in small sample sizes (Chen et al., 2018; Grady et al., 2021; Marek et al., 2022). The main cause of fMRI’s limited reproducibility relies on the blood-oxygen-level-dependent (BOLD) signal. The BOLD signal has a low signal-to-noise ratio (Yakushev et al., 2017; Verger and Guedj, 2018), and several repetitions of the same fMRI protocol are necessary to increase the sensitivity of measurements. In addition, the signal-to-noise ratio may vary drastically between fMRI runs, degrading the robustness and reproducibility of this method (Cheng, 2012). Moreover, the neurovascular coupling—alterations in local perfusion that occur in response to neuronal activity changes— can heavily affect the BOLD signal (Yakushev et al., 2017; Verger and Guedj, 2018; Sala and Perani, 2019; Carli et al., 2021). In this context, [18F]FDG-PET brain connectivity imaging may provide advantages to fMRI. [18F]FDG-PET signal originates mainly from excitatory synaptic activity, localized in gray matter tissue, thus, being inherently less dependent on neurovascular coupling than the BOLD signal (Wehrl et al., 2013). Moreover, [18F]FDG-PET benefits from better signal-to-noise ratios and out-of-sample replications than fMRI, contributing to the robustness and reproducibility of [18F]FDG-PET findings. Indeed, [18F]FDG-PET connectivity measures are reported as robust, specific, and reproducible also in a small sample of patients (Schindlbeck and Eidelberg, 2018; Ripp et al., 2020). Thus, applying metabolic connectivity approaches to [18F]FDG-PET data provides a comprehensive approach to deciphering changes in brain networks in neurodegenerative diseases (Carli et al., 2021). Few studies have explored the large-scale network with metabolic connectivity in LB diseases. PD is characterized by a connectivity derangement in the large-scale frontal networks, including the motor one (Sala et al., 2017). DLB metabolic connectivity showed a severe involvement of the posterior cortical, limbic, and attention networks, strongly related to the heterogeneous clinical symptoms (Sala et al., 2019).

Despite the above-described large-scale brain network alterations in PD and DLB separately, there is a lack of comprehensive studies on the LB spectrum, including the prodromal phase (iRBD). This study investigates shared and disease-specific changes in the large-scale networks (resting-state networks, RSNs), in three clinical groups (iRBD, PD without cognitive deterioration over time, and DLB) throughout the brain metabolic connectivity approach. Due to this, we evaluated metabolic connectivity changes by applying a seed-based interregional correlation analysis (IRCA).

## Materials and methods

### Participants

#### Subjects with isolated rapid eye movement sleep behavior disorder

Thirty subjects with a polysomnography-confirmed diagnosis of iRBD (American Academy of Sleep Medicine, 2014) were retrospectively selected from the clinical database of the Sleep disorders Centre of Turro San Raffaele Hospital, Milan, Italy.

#### Patients with stable Parkinson’s disease

Twenty-eight patients diagnosed with PD without cognitive impairment at baseline and follow-up (8 years) were retrospectively selected from the clinical and imaging database of the Neurology Unit, Department of Clinical and Experimental Sciences, at the University of Brescia, Brescia, Italy. The clinical diagnosis of PD was made according to the Movement Disorder Society Clinical Diagnostic Criteria (Postuma et al., 2015).

#### Patients with dementia with Lewy bodies

Thirty patients with a diagnosis of probable DLB (McKeith et al., 2017) were retrospectively selected from the clinical and imaging database of San Raffaele Hospital, Milan, Italy.

#### Healthy controls

Thirty HC were randomly selected for comparison from the internal database of the In Vivo Human Molecular and Structural Neuroimaging Unit, IRCCS San Raffaele Scientific Institute, Milan, Italy. They did not show a medical history of neurological or psychiatric diseases or other chronic illnesses and were not taking psychoactive medication.

All participants or their informed caregivers provided informed consent. The protocols conformed to the Ethical Standards of the Helsinki Declaration for the protection of human subjects.

### [18F]FDG-PET image acquisition and pre-processing

All subjects underwent an [18F]FDG–PET imaging session. [18F]FDG–PET scans were performed by a General Electric Discovery LS PET/CT or a multi-ring General Electric Discovery. The [18F]FDG–PET acquisition procedures conformed to the European Association of Nuclear Medicine guidelines (Varrone et al., 2009). Static emission images were acquired 45 min after injecting 185–250 MBq of [18F]FDG *via* a venous cannula, with a 15-min scan duration. Data obtained from steady-state static [18F]FDG–PET acquisition were demonstrated to be comparable to the [18F]FDG–PET data obtained from dynamic quantitative acquisition procedures (Signorini et al., 1999). All images were reconstructed using an ordered subset-expectation maximization algorithm. Attenuation correction was based on CT scans.

Image pre-processing was performed using the SPM12 software running in Matlab (MathWorks Inc., Sherborn, MA, United States). First, each [18F]FDG–PET image was spatially normalized to a specific [18F]FDG–PET template in the MNI space (Della Rosa et al., 2014). Images were spatially smoothed with an isotropic 3D Gaussian kernel (Full width at half maximum FWHM: 8-8-8 mm). Global mean scaling was applied to each image to account for between-subject uptake variability (Perani et al., 2014).

### Metabolic connectivity of the resting-state networks analysis

#### Interregional correlation analysis

We performed IRCA using a voxel-wise SPM procedure (Lee et al., 2008) to investigate the metabolic connectivity of the RSNs in the three groups of patients (iRBD, DLB, and PD) as compared to HC. The central assumption of this analysis dates back to the work by Horwitz et al. (1984), demonstrating that brain regions whose glucose metabolism is correlated at rest are functionally associated.

IRCA is a voxel-wise approach previously validated for [18F]FDG-PET data that allows the derivation of RSNs from relevant seed regions selected based on previous literature (Horwitz et al., 1984; Lee et al., 2008). It relies on selecting a region of interest (ROI), or seed, from which the average tracer uptake value is extracted. The correlation between average uptake in the seed and uptake in each voxel in the rest of the brain is then tested (Lee et al., 2008) to estimate the connectivity profile, or connectivity map, of the seed of interest. Of note, brain networks estimated using [18F]FDG-PET data benefit from disease specificity and robustness also in a small sample of patients (*N* ≥ 20) (Schindlbeck and Eidelberg, 2018; Ripp et al., 2020). In this study, we applied IRCA analysis in three groups of patients and one HC cohort, ensuring a reliable number of subjects per group (*N* ≥ 25). In addition, to increase the reliability and reproducibility of RSNs, we considered the seeds of RSNs previously validated. Specifically, we considered the RSNs affected in PD and DLB, following the previous literature (Caminiti et al., 2017; Sala et al., 2017, 2019; Iaccarino et al., 2018), namely, the anterior and posterior default mode networks (ADMN and PDMN), the attentive network (ATTN), the frontal executive network (EXN), the limbic network (LIN), the motor network (MN), the somatosensory network (SN), the primary, and high visual networks (PVN and HVN; details on the selected ROIs are provided in Table 1). We used the REX toolbox^1^ to extract the mean metabolism values from nine ROIs (seeds), entering as sources the scaled [18F]FDG-PET images of patients and HC. Then, the average seed uptake was set as a variable of interest in a multiple regression model in SPM12, entering age and gender as nuisance covariates for each network. The statistical threshold was set at *p* = 0.001, with Kep ≥ 100 voxels.

**TABLE 1:** Selected ROIs for the analysis.

	Seeds	Derivation atlases
ADMN	Ventromedial frontal cortex	Shirer 2012 Functional RSNs atlas
ATTN	Angular gyrus, supramarginal gyrus	Shirer 2012 Functional RSNs atlas
EXN	Dorsolateral prefrontal cortex	Sallet’s Dorsal Frontal Parcellation atlas
HVN	Inferior occipital cortex, middle occipital cortex	Shirer 2012 Functional RSNs atlas
LIN	Amygdala	Anatomical Automatic Labeling (AAL) atlas
MN	Precentral gyrus	Anatomical Automatic Labeling (AAL) atlas
PDMN	Posterior cingulum, precuneus	Shirer 2012 Functional RSNs atlas
PVN	Calcarine cortex	Shirer 2012 Functional RSNs atlas
SN	Postcentral gyrus	Anatomical Automatic Labeling (AAL) atlas

ADMN, anterior default mode network; ATTN, attentive network; EXN, executive network; HVN, high visual network; LIN, limbic network; MN, motor network; PDMN, posterior default mode network; PVN, primary visual network; SN, somatosensory network.

#### Connectivity comparison and metrics

First, we aimed to evaluate the integrity of each RSN. To do that, we compared each clinical group with HC (iRBD vs. HC, PD vs. HC, and DLB vs. HC). We quantified the RSN extension and the overlap between each clinical group and HC. The difference in RSN extension between patients and HC may reflect two different and potentially malignant mechanisms: increased or decreased metabolic connectivity of the seed with a set of brain areas compared to healthy brain functioning. Then, the comparison of spatial topography aimed to evaluate if the set of areas metabolically connected to the seed is similar or different in patients and HC. We used well-established and validated connectivity metrics to quantify these differences. Differences in network extension and topography were measured according to the number of correlated voxels (Ballarini et al., 2016; Galbiati et al., 2021) and Dice (DC) similarity coefficient index (Savio et al., 2017), respectively.

When we found alterations compared to HC, we also evaluated the commonalities/differences of the pathological changes among the clinical spectrum (i.e., iRBD, PD, and DLB). To do that, we performed direct comparisons between clinical groups (i.e., iRBD vs. PD, iRBD vs. DLB, PD vs. DLB). Specifically, we evaluated the topographical differences, also considering the deviation from HC of each group, using a previously validated metabolic connectivity index: weighted DC coefficient (wDC; Carli et al., 2020c; Mencarelli et al., 2020). Compared to the standard unweighted DC coefficient, the wDC provides a similarity index that considers not only spatial similarity between groups of patients but also the similarity of the connectivity changes that emerged in comparison with HC.

The following sub-paragraphs explain in detail the above-mentioned metabolic connectivity metrics.

##### Network spatial extension (voxel count)

We extracted the number of seed-correlated voxels for each considered network in patients and control groups to quantify the degree and the direction of differences in the spatial extension of the metabolic connectivity (Ballarini et al., 2016; Galbiati et al., 2021). Specifically, computing the difference (Δ) between HC and clinical groups for each RSN for obtaining the degree (absolute value of Δ) and the direction of spatial differences (the sign of Δ, positive = increase and negative = decrease).

##### Network topography (Dice coefficient and weighted Dice coefficient)

Changes in RSNs connectivity between clinical groups and HC were assessed with the DC and computed using FSL software (Jenkinson et al., 2012). DC measures similarity between large-scale networks by computing the normalized amount of their overlap (i.e., volume overlap between two binary masks divided by their mean volume) (Savio et al., 2017). To evaluate the differences and similarities among clinical groups (i.e., iRBD vs. PD, iRBD vs. DLB, and PD vs. DLB), we computed the weighted wDC (Carli et al., 2020c; Mencarelli et al., 2020) to account for their deviation from HC RSNs. Compared to the standard unweighted DC coefficient, the wDC provides a similarity index that advantageously takes into account not only spatial similarity but also the similarity of the connectivity signs [i.e., positive (higher connectivity than controls) and negative (lower connectivity than controls)]. To do so, we derived a ratio of the voxel count difference (SRΔ) between HC and clinical groups for each RSN:


(1) $$\text{SR}\,\Delta =\frac{\left(\mathrm{Voxel}\,\mathrm{count}\,\mathrm{clinical}\,\mathrm{group}\,\mathrm{RSN}\right)-\left(\mathrm{Voxel}\,\mathrm{count}\,\mathrm{HC}\,\mathrm{RSN}\right)}{\left(\mathrm{Voxel}\,\mathrm{count}\,\mathrm{HC}\,\mathrm{RSN}\right)}$$

Positive and negative ratios represented the increase and decrease of connectivity compared to HC in terms of spatial network extension. In other words, the SRΔ > 0 indicates that patients showed a higher extension of a specific RSN than HC, and SRΔ < 0 reflected a lower extension. In this way, the product sign reflects whether the groups had the same (both increase or decrease connectivity, positive sign) or different (one increase and the other decrease, or vice versa, negative sign) connectivity changes compared to HC. To obtain the wDC, we added the product of SRΔ of the two groups to the DC. For example, we did a linear computation to calculate the wDC for iRBD and PD groups; we first obtained the traditional DC, and then we added the product of SRΔs computed for iRBD and PD groups to DC. The wDC is a data-based similarity coefficient that measures similarity relative to the specific dataset investigated. Thus, this procedure quantifies the degree of similarity in connectivity alterations among the different clinical groups accounting for the connectivity networks’ spatial changes compared to HC. Expressly, wDC values below or close to 0 indicated null or very low similarity between the two groups.

The wDC is a relatively new index that can be applied in different ways and to different imaging modalities (Carli et al., 2020c; Mencarelli et al., 2020). Thus, we aimed to test whether the here computed index effectively measured the topographical differences between clinical groups, considering the deviation from HC in terms of RSNs extension. The wDC is supposed to be higher when two groups of patients have a high percentage of topographical overlap (measured through the traditional DC), and/or both groups showed the same direction of changes compared to HC (increased or decreased network extension in comparison to HC). To verify that, we applied a ranking strategy. For each comparison (iRBD vs. PD, iRBD vs. DLB, and PD vs. DLB), we classified the nine RSN as concordant (both groups have increases/decreases) or discordant (one group has increases and the other decreases, or vice versa) in terms of extension compared to HC. For each comparison (iRBD vs. PD, iRBD vs. DLB, and PD vs. DLB), we also classified the RSNs with moderate-to-good overlap (DC > 0.40) or fair/poor overlap (DC < 0.40), according to the literature guidelines (Savio et al., 2017). Then, we defined a network *similar* when two groups showed either concordant direction of extension changes compared to HC or a moderate degree of overlap in DC. We defined a network *not similar* when it had discordant changes in extension compared to HC and fair/poor topographical overlap. Last, we tested whether the wDC was statistically different between similar and not similar RSNs for each comparison using ANOVA statistical models.

## Results

The demographic and clinical features of the study groups are reported in Table 2. Notably, PD patients were significantly younger than iRBD and DLB (*p* < 0.05), whereas MMSE excluded significant cognitive changes in iRBD and PD compared to DLB (*p* < 0.05). Thus, the iRBD cohort was characterized by the absence of relevant cognitive impairments (MMSE _*mean* ± SD_ = 27.46 ± 2.65) and motor signs (UPDRS III_*mean* ± SD_ = 0.54 ± 1.2). At the same time, 97% of the DLB group featured parkinsonism.

**TABLE 2:** Demographic and clinical features of the samples.

	iRBD	PD	DLB	HC	Statistic
*N*	30	28	30	30	
Gender (F/M)	2/28	12/16	13/17	13/17	*p* = 0.002^1a,b,e^
Age, y (Mean ± SD)	70.07 ± 6.82	62.68 ± 10.82	70.83 ± 8.45	63.03 ± 2.8	*p* = 0.000^2a,c,d,e^
Education, y (Mean ± SD)	11.1 ± 4.79	8.25 ± 4.26	9.37 ± 4.14	–	*p* = 0.053^2^
Disease duration, y (Mean ± SD)	5.33 ± 3.12	4.25 ± 2.59	2.34 ± 2.12	–	*p* = 0.000^2b,c^
MMSE, corrected score (Mean ± SD)	27.46 ± 2.65	28.71 ± 1.49	17.31 ± 4.59	–	*p* = 0.000^2b,c^

^1^Chi-squared test.

^2^One-way ANOVA.

Significant differences at *post hoc* comparisons, significant at *p* < 0.05, using Bonferroni-correction for multiple comparisons:

^a^iRBD ≠ PD.

^b^iRBD ≠ DLB.

^c^PD ≠ DLB.

^d^DLB ≠ HC.

^e^iRBD ≠ HC.

iRBD, isolated REM sleep behavior disorder; PD, Parkinson’s disease; DLB, dementia with Lewy Bodies; N, number; y, year; F, female; M, Male; MMSE, Mini Mental State Examination; SD, standard deviation.

### Interregional correlation analyses results

#### Comparisons between clinical groups and healthy controls

##### Isolated REM sleep behavior disorder subjects vs. healthy controls

The MN (DC = 0.19; Δvoxel = -7,543), PDMN (DC = 0.29; Δvoxel = 8,717), and ATTN (DC = 0.39; Δvoxel = 7,463) were the most altered networks in iRBD. Specifically, MN seed (i.e., precentral gyrus) lost metabolic connectivity mainly with the bilateral supplementary motor area and superior frontal gyrus. PDMN and ATTN showed instead increased connectivity compared to HC. In PDMN, the seeds (i.e., posterior cingulum and precuneus) showed increased metabolic connectivity with the angular gyrus, cuneus, precuneus, and superior frontal gyrus, bilaterally. In ATTN, the seed showed increased connectivity with the angular gyrus, posterior cingulum, and middle temporal gyrus, bilaterally.

**FIGURE 1:**
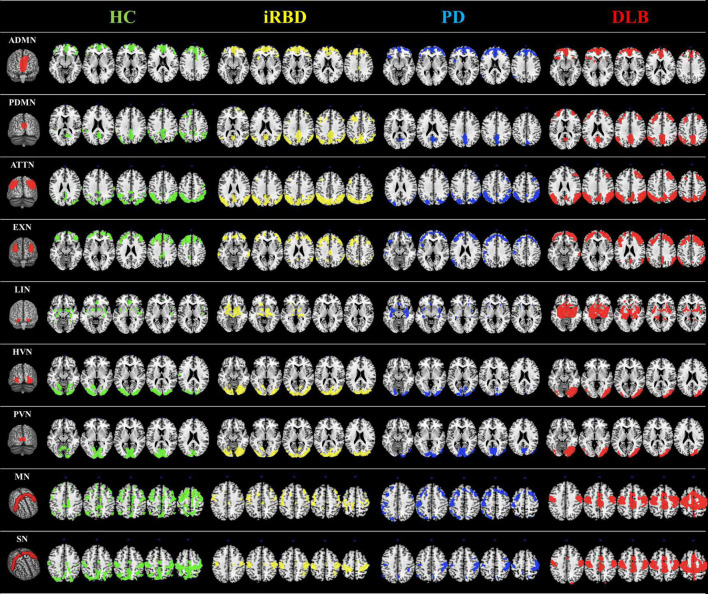
RSNs analysis. RSNs topography in HC (green overlaid to the anatomical template), iRBD (yellow overlaid to the anatomical template), PD (blue overlaid to the anatomical template), and DLB (red overlaid to the anatomical template) for the ADMN, PDMN, ATTN, EXN, LIN, HVN, PVN, MN, and SN. The selected seed for each network is shown in the first column. A connectivity derangement is present in all the RSNs. RSNs were obtained using seed-based intercorrelation analysis. Clusters with a minimum spatial extent of 100 voxels are shown, with a voxel-wise significant threshold of *p* = 0.001. iRBD, isolated REM sleep behavior disorder; PD, Parkinson’s disease; DLB, dementia with Lewy Bodies; HC, healthy controls; ADMN, anterior default mode network; ATTN, attentive network; EXN, executive network; HVN, high visual network; LIN, limbic network; MN, motor network; PDMN, posterior default mode network; PVN, primary visual network; SN, somatosensory network.

**FIGURE 2:**
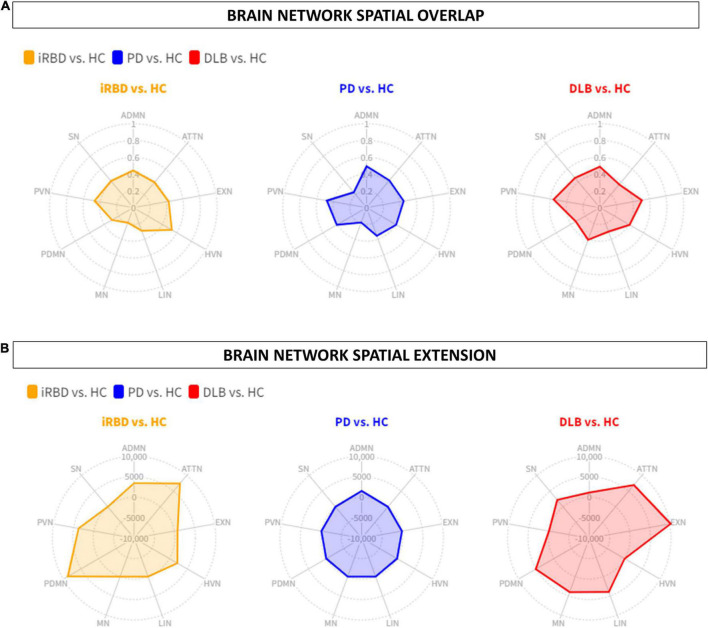
Brain networks spatial overlap and extension. **(A)** The spatial overlap between HC and iRBD (orange), PD (blue), and DLB (red) in the ADMN, PDMN, ATTN, EXN, LIN, HVN, PVN, MN, SN, is expressed by DC. **(B)** The spatial extension of all networks in RBD (orange), PD (blue), and DLB (red) compared to HC, expressed as the difference between HC and each clinical group (Δ). iRBD, isolated REM sleep behavior disorder; PD, Parkinson’s disease; DLB, dementia with Lewy Bodies; HC, healthy controls; ADMN, anterior default mode network; ATTN, attentive network; EXN, executive network; HVN, high visual network; LIN, limbic network; MN, motor network; PDMN, posterior default mode network; PVN, primary visual network; SN, somatosensory network.

The other RSNs showed moderate differences compared to HC in terms of topography extension and overlap. The ADMN (DC = 0.44; Δvoxel = 3449), EXN (DC = 0.43; Δvoxel = 799), HVN (DC = 0.53; Δvoxel = 2244), and PVN (DC = 0.47; Δvoxel = 3775) showed an increase of metabolic connectivity compared to HC. On the other hand, SN (DC = 0.41; Δvoxel = -7,151) and LIN (DC = 0.29; Δvoxel = -1,005) showed a decrease of metabolic connectivity in comparison to HC. See Figures 1, 2 for the graphical representation.

##### Parkinson’s disease patients vs. healthy controls

The MN (DC = 0.18; Δvoxel = -5,846) and SN (DC = 0.23; Δvoxel = -9,561) were the most altered networks in PD patients, both characterized by a decrease of connections compared to HC. Specifically, MN seed extensively lost metabolic connections mainly with the bilateral supplementary motor area and middle and superior frontal gyrus. In SN, the seed (i.e., bilateral postcentral gyrus) showed a widespread connectivity decrease with inferior and superior parietal gyrus, precuneus, and paracentral lobule.

The other RSNs showed moderate differences compared to HC in terms of topography extension and overlap. The ADMN (DC = 0.50; Δvoxel = 1,552), EXN (DC = 0.45; Δvoxel = -3,061), HVN (DC = 0.41; Δvoxel = -4,675), PVN (DC = 0.48; Δvoxel = -2,259), PDMN (DC = 0.41; Δvoxel = -3,035), ATTN (DC = 0.42; Δvoxel = -1,068), and LIN (DC = 0.36; Δvoxel = -1,632). See Figures 1, 2 for the graphical representation.

##### Dementia with Lewy bodies patients vs. healthy controls

The LIN (DC = 0.30; Δvoxel = 3,988), PDMN (DC = 0.33; Δvoxel = 5,136), and ATTN (DC = 0.36; Δvoxel = 6,963) were the most altered networks in DLB patients, all characterized by an increase of metabolic connectivity compared to HC. Specifically, LIN seed (i.e., amygdala) showed increased metabolic connectivity mainly with the bilateral hippocampus, parahippocampus, and middle temporal pole. In PDMN, we found increased metabolic connectivity between the posterior seed and frontal brain regions (i.e., bilateral middle frontal gyrus and superior frontal gyrus). Similarly, in ATTN, IRCA revealed increased connectivity between posterior and anterior regions. The ATTN seed (i.e., angular gyrus and supramarginal gyrus) showed increased metabolic connectivity, mainly with the right middle frontal gyrus and middle orbital frontal cortex. The seed showed higher connectivity with the posterior portion of the middle temporal gyrus bilaterally.

The other RSNs showed moderate differences compared to HC in terms of spatial extension and overlap. The ADMN (DC = 0.48; Δvoxel = 1,138), EXN (DC = 0.51; Δvoxel = 10,233), MN (DC = 0.41; Δvoxel = 4,027), and SN (DC = 0.46; Δvoxel = 2,225). Only the visual networks, PVN (DC = 0.56; Δvoxel = -2,876) and HVN (DC = 0.41; Δvoxel = -1,424), showed a reduction of metabolic connectivity compared with controls. See Figures 1, 2 for the graphical representation.

### Direct comparisons between clinical groups

**FIGURE 3:**
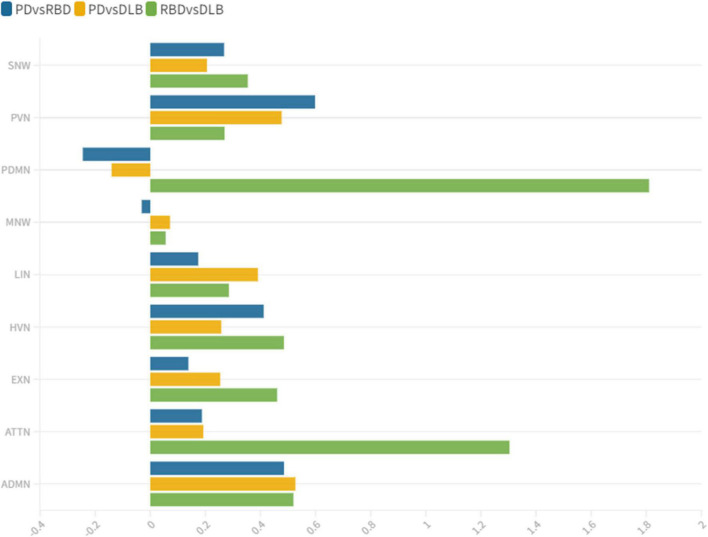
wDC similarity coefficient among clinical groups. wDC for every RSN is shown. iRBD and DLB (green) showed the highest wDC values, especially in PDMN and ATTN. iRBD, isolated REM sleep behavior disorder; PD, Parkinson’s disease; DLB, dementia with Lewy Bodies; ADMN, anterior default mode network; ATTN, attentive network; EXN, executive network; HVN, high visual network; LIN, limbic network; MN, motor network; PDMN, posterior default mode network; PVN, primary visual network; SN, somatosensory network.

**FIGURE 4:**
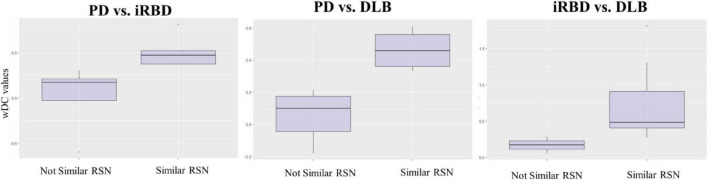
wDC values in similar and not similar RSNs. The figure represents the mean values of wDC in RSN classified as *similar* and *not similar* in iRBD vs. PD, PD vs. DLB, and RBD vs. DLB comparison (from right to left).

See Table 3 and Figures 1, 3, 4 for wDC and DC values and their graphical representation.

**TABLE 3:** wDC coefficient for comparison in clinical groups.

	wDC PD vs. IRBD	wDC DLB vs. PD	wDC IRBD vs. DLB
ADMN	0.52	0.54	0.52
ATTN	0.18	0.18	1.30
EXN	0.30	0.21	0.46
HVN	0.16	0.37	0.48
LIN	0.37	0.33	0.29
MN	0.47	–0.04	0.06
PDMN	–0.60	–0.18	1.81
PVN	0.37	0.61	0.27
SN	0.81	0.10	0.35

iRBD, isolated Rem sleep behavior disorder; PD, Parkinson’s disease; DLB, dementia with Lewy bodies; WDC, weighted Dice coefficient; ADMN, anterior default mode network; ATTN, attentive network; EXN, executive network; HVN, high visual network; LIN, limbic network; MN, motor network; PDMN, posterior default mode network; PVN, primary visual network; SN, somatosensory network.

#### Isolated REM sleep behavior disorder subjects vs. Parkinson’s disease patients

iRBD and PD patients showed an overall low degree of overlap in RSNs metabolic reconfiguration, with most values close to 0 (wDC ranging from -0.60 to 0.81). According to the ranking strategy, PD and iRBD showed five similar RSNs (i.e., ADMN, LIN, MN, PVN, SN) and four dissimilar RSNs (i.e., ATTN, EXN, HVN, and PDMN). Similar and dissimilar RSNs showed a significant difference in wDC mean values (similar mean ± SD: 0.51 ± 0.18; not similar mean ± SD: 0.01 ± 0.41; *p* = 0.016). The PDMN was the network where the two groups showed the lowest similarity value (wDC = -0.60). The highest level of similarity was found in SN (wDC = 0.81), where both groups showed a decrease in network extension compared to HC.

#### Isolated REM sleep behavior disorder subjects vs. dementia with Lewy bodies patients

iRBD patients showed an overall high degree of overlap with DLB patients in RSNs metabolic reconfigurations (wDC ranging from 0.06 to 1.81). According to the rank strategy, in DLB and iRBD, most RSNs were similar (i.e., ADMN, ATTN, EXN, HVN, PDMN PVN, and SN), and only two were not similar RSNs (i.e., LIN and MN). Similar (mean ± SD: 0.75 ± 0.58) RSNs showed slightly higher wDC compared to dissimilar (mean ± SD: 0.17 ± 0.16) RSNs without reaching the statistical significance in wDC (*p* = 0.111), further demonstrating a high degree of similarity between iRBD and DLB. The highest similarities were found in PDMN (wDC = 1.81) and ATTN (wDC = 1.30), where both DLB and iRBD networks showed increased connectivity between frontal and posterior regions. Accordingly, both groups showed an increased extension of these two networks compared to HC, with increased anterior–posterior connectivity.

#### Parkinson’s disease patients vs. dementia with Lewy bodies patients

DLB and PD patients showed an overall low degree of overlap in RSNs metabolic reconfigurations, with all the values close to 0 (wDC ranging from -0.18 to 0.61). PD and DLB showed four similar RSNs (i.e., ADMN, HVN, LIN, and PVN) and five dissimilar RSNs (i.e., ATTN, EXN, MN, PDMN, and SN) with significant differences in wDC mean values (similar mean ± SD: 0.46 ± 0.13; not similar mean ± SD: 0.05 ± 0.16; *p* = 0.016). The PDMN was the network where they showed the lowest overlap value (wDC = -0.18). The PD group showed a decreased extension in this network compared to HC; an increased network extension characterized the DLB cohort. From a topographical point of view, PD patients showed functional isolation of the seed that lost its metabolic connection with the brain, and the DLB cohort showed recruitment of frontal cortical regions, especially on the right side.

## Discussion

This study assessed the large-scale brain network integrity in the spectrum of LB diseases, giving a comprehensive picture of connectivity reconfiguration from the prodromal to the overt clinical phases. We found altered connectivity patterns in the majority of the multiple large-scale brain networks, with interesting overlaps and differences within the disease spectrum. Distinct connectivity reconfigurations characterized the three clinical groups (iRBD, PD stable, and DLB). The SN and MN were the most severely affected networks in PD, and the LIN, ATTN, and PDMN in the DLB cohort. These specific patterns of connectivity reconfigurations might represent the dysfunctional substrates underlying the different clinical phenotypes. PD patients showed mainly motor symptoms (UPDRS III_*mean* ± SD_ = 15.04 ± 7.02) and stable cognitive progression over time (no dementia evidence during 8 years of follow-up after the first clinical diagnosis). The SN and MN play a central role in preparing and executing the motor function (Tessitore et al., 2019). Their connectivity in PD patients is frequently reported as disrupted (Caspers et al., 2021; Chen et al., 2021), and these functional changes are correlated with the motor symptoms’ severity progression (Kojovic et al., 2012). In DLB, metabolic connectivity alterations showed a severe involvement of PDMN, LN, and ATTN, consistently with the pathological and clinical heterogeneity of this condition (Iaccarino et al., 2018; Sala et al., 2019). In particular, PDMN and ATTN are associated with visual hallucination (Sala et al., 2019), which were present as core symptoms in 73% (22 out of 30) of our DLB cohort. Notably, iRBD already showed severe alterations in PDMN and ATTN networks compared to HC, sharing the same connectivity reconfiguration with DLB. On the contrary, iRBD and PD stable showed an overall low degree of similarity in metabolic connectivity reconfiguration in all the large-scale brain networks; in PDMN, the two groups showed the most prominent differences—likewise PD and DLB– (Figure 3). These results support that LB diseases are brain multisystem disorders, dynamically involving abnormal functioning of most large-scale networks, already in the prodromal phases. The high similarities between iRBD and DLB metabolic connectivity changes, together with the differences between PD and both iRBD and DLB, support the iRBD condition as a red flag for a progression to the severe phenotypes of alpha-synucleinopathies.

In the iRBD group, all networks showed a metabolic connectivity reconfiguration compared to HC, with the overlap ranging from poor to moderate (Figure 2A). MN, PDMN, and ATTN were the most altered networks. Connectivity alterations of the MN are in line with previous fMRI connectivity data (Ellmore et al., 2013; Rolinski et al., 2016; Yamada et al., 2019; Wakasugi et al., 2021). These fMRI studies report functional connectivity abnormalities in movement-related brain networks in iRBD patients, including sensory-motor networks. Rolinski et al. (2016) demonstrated that functional alteration of motor-related brain networks (basal ganglia network) is already present in iRBD and comparable to clinically manifested PD patients, despite the spared dopaminergic innervation. Thus, this study demonstrated that functional connectivity could be considered a sensitive measure to early brain changes, identifying brain alterations before a detectable neurotransmission impairment. A recent study further confirmed that motor-related networks (basal ganglia and sensory-motor networks) are dysfunctional in patients with iRBD without manifest motor deficits (Wakasugi et al., 2021). In this study, we found a severe impairment of motor-related brain networks detected with metabolic connectivity measures for the first time (Figure 1). Thus, our findings, together with previous fMRI evidence, demonstrated the presence of early dysfunction in the motor large-scale brain networks in iRBD patients even before the onset of clinically relevant motor symptoms and dopaminergic impairments.

Concerning the PDMN and ATTN, we found increased connectivity in iRBD patients compared to HC. Of note, iRBD showed a high overlap of metabolic connectivity reconfiguration in these networks with DLB patients, who showed increased anterior-posterior metabolic connections as well (Figures 1, 2B, 3). These functional connectivity alterations related to posterior parietal regions agree with recent fMRI findings (Campabadal et al., 2020). Campabadal et al. (2020) also found a correlation between functional connectivity in temporoparietal regions and cognitive performance in iRBD, suggesting a cognitive role of the derangement in posterior brain networks. In [18F]FDG-PET studies—similar to fMRI data—connectivity changes are often explained in terms of function. Decreased connectivity indicates the functional separation between regions, whereas increased connectivity indicates increased functional pairing (Pievani et al., 2014; Sala and Perani, 2019). Increased connectivity may indicate a “beneficial” compensatory process when increased connectivity affects metabolically spared brain regions. Thus, the recruitment of functionally spared brain regions might help cope with neurodegeneration. Most iRBD patients show hypometabolic patterns characterized by occipitoparietal hypometabolism with a sparing of frontal regions (Meles et al., 2018; Carli et al., 2020b), resembling those of DLB patients (Caminiti et al., 2019). In this context, the here reported increased functional connectivity between posterior regions and frontal brain area might represent compensation for the underlying neurodegeneration process in iRBD. Indeed, in DLB patients, we found the same compensatory metabolic connectivity signature, confirming previous results (Caminiti et al., 2017; Sala et al., 2019; Carli et al., 2020c). Also, the visual networks, PVN and HVN, showed increased connectivity between the seed and occipital regions compared to HC (Figures 1, 2). Conversely, DLB patients showed reduced metabolic connectivity within the same large-scale brain networks. Increased occipital connectivity in iRBD aligns with previous fMRI findings (Ellmore et al., 2013; Byun et al., 2020) and can support the recently proposed hypothesis about the cholinergic compensation to occipital damage in these patients (Bedard et al., 2019; Byun et al., 2020). Previous studies found that iRBD’s cognitive performance positively correlates with functional measures of increased connectivity, indicating a possible ongoing compensation process linked to occipital regions (Byun et al., 2020; Campabadal et al., 2020). Moreover, recent molecular data (PET neuroimaging with the 18F-fluoroethoxybenzovesamicol) demonstrated for the first time that the brain cholinergic alterations in patients with iRBD are characterized by increased cholinergic innervation in multiple brain areas (Bedard et al., 2019), suggesting an ongoing compensatory cholinergic upregulation. The posterior occipital regions are crucial pathophysiological regions in cognitive decline in DLB and PDD (Pilotto et al., 2018; Caminiti et al., 2019). Indeed, in our study, DLB patients showed disrupted HVN and PVN metabolic connectivity connections. We hypothesize that increased occipital metabolic connectivity in iRBD patients may be a first protective compensatory response to early synucleinopathy-related changes in the occipital region, which is subsided by a connectivity decrease in DLB due to the advancement of the neurodegenerative processes and further accumulation of pathology.

In PD patients, we found a loss of connectivity mainly affecting the SN and MN networks showing a poor overlap with HC (Figures 1, 2). These networks play a central role in motor function (Tessitore et al., 2019) and are usually disrupted in PD patients (Caspers et al., 2021; Chen et al., 2021), leading to the motor symptoms’ progression (Kojovic et al., 2012). A recent multimodal imaging study demonstrated the close relationship between dopaminergic depletion, degeneration of nigrostriatal projection, and the destruction of sensory-motor networks in PD, mainly driven by the putamen’s functional impairment (Ruppert et al., 2020). In addition to SN and MN, we also found a decreased connectivity in ATTN, EXN, and PDMN, although less severe. These findings are consistent with previous metabolic connectivity data in PD (Sala et al., 2017). PDMN showed a loss of connectivity between the seed and the anterior and posterior components. PDMN disruption was a direct consequence of PD pathology (Yao et al., 2014) and is closely associated with PD dopaminergic depletion (Spetsieris et al., 2015). Overall, the main connectivity alterations in PD are possibly led by the dopaminergic dysfunction characterizing this clinical entity. According to the dual syndrome hypothesis, the frontostriatal dysfunction is a characteristic of non-demented PD patients, as our cohort, and the posterior cortical dysfunction, found here in DLB and iRBD, is considered a signature of cognitive decline (Kehagia et al., 2013). Consistently, we did not find a posterior connectivity alteration in PD, resembling a DLB pattern, as an early predictor of dementia. The frontal executive connectivity derangement seems to be disease-specific for stable PD, profoundly differing from connectivity alterations of iRBD and DLB.

In DLB patients, metabolic connectivity derangement involved PDMN, ATTN, and LIN, consistent with previous evidence (Sala et al., 2019). DLB connectivity alterations in large-scale RSNs are also strongly related to clinical symptoms (Sala et al., 2019). The alterations of PDMN, ATTN, and visual networks are associated with visual hallucinations (Iaccarino et al., 2018; Sala et al., 2019). The biological explanation for these findings points to cholinergic damage, leading to the breakdown of the functional relationship among the calcarine cortex, lateral occipital cortex, and parietal cortex (Klein et al., 2010). In our DLB group, both visual networks—PVN and HVN—showed decreased metabolic connectivity compared to HC (Figures 1, 2B). On the other hand, PDMN and ATTN were characterized by abnormal connectivity increases involving the dorsolateral prefrontal cortex. As discussed above in iRBD, the increased poster-anterior connectivity can be interpreted as a compensative mechanism. In DLB patients, cognitive reserve proxies (i.e., education and specific occupational profiles) modulate both ATTN and PDMN throughout neural compensation mechanisms (Carli et al., 2020a). Specifically, it has been demonstrated that highly educated DLB can engage the anterior brain regions to cope with posterior pathology. However, in overt DLB, the dorsolateral prefrontal cortex can be part of the hypometabolic pattern (Caminiti et al., 2019). When increased connectivity affects metabolically impaired brain regions, it may represent a form of maladaptive functional reorganization resulting from the brain’s failure to cope with the damage. For instance, the proper one-to-one connection in healthy brains can be substituted by a widespread dysfunctional connectivity pattern toward several regions resulting in maladaptive connectivity increases. However, separating beneficial from maladaptive processes remains a challenge in need of longitudinal data and correlation with behavior (Schoonheim, 2017). Regarding LIN alterations in DLB, previous evidence showed that these are associated with the presence of clinical RBD (Sala et al., 2019). Notably, the LIN seed is the amygdala which is considered an early site of LB accumulation (Braak et al., 2003), and amygdala LB pathology has been associated with visual hallucinations (Harding and Halliday, 2001).

Overall, despite large-scale brain network alterations in all clinical groups, high topographical overlaps were found only in iRBD and DLB, and not in PD. Specifically, the involvement of the posterior and limbic networks appears as the DLB hallmark related to the clinical core symptoms, such as visual hallucinations (Sala et al., 2019). These RSN alterations already in the iRBD phase may represent an early signature of future clinical progression. Similarly, the high spatial overlap in the ATTN between iRBD and DLB can be related to attentional and executive deficits in the two syndromes. Cognitive tests assessing attention and executive functions strongly predict conversion to dementia in RBD patients (Génier Marchand et al., 2018).

In conclusion, our findings revealed that metabolic connectivity alterations in large-scale networks are already present in the iRBD phase. Strong connectivity similarities emerged between iRBD and DLB, showing comparable metabolic patterns in RSNs relevant for cognitive decline. Differently, PD’s altered connectivity patterns support its more benign phenotype without dementia development. Thus, our findings showed the different RSNs derangement underlying the spectrum conditions. The disease-specific vulnerabilities shared by iRBD and DLB indicate that iRBD can be considered a risk factor for progression to a malignant alpha-synucleinopathy phenotype (Berg et al., 2021).

## Data availability statement

The raw data supporting the conclusions of this article will be made available by the authors, without undue reservation.

## Ethics statement

The studies involving human participants were reviewed and approved by the IRCCS San Raffaele Scientific Institute, Milan, Italy and University Hospital ‘Spedali Civili’, Brescia, Italy. The patients/participants provided their written informed consent to participate in this study.

## Author contributions

CB, GC, and DP: study concept and design and drafting and revising the manuscript. CB, EB, and GC: analysis and interpretation of data and drafting and revising the manuscript. APa, AG, APi, and LF-S: acquisition of data and revising the manuscript. All authors revised and approved the final version of the manuscript.

## Footnotes

1
https://www.nitrc.org/projects/rex/
